# Supplementary material for: ICER is requisite for Th17 differentiation
Source: Nat Commun. 2016 Sep 29;7:12993. doi: 10.1038/ncomms12993 (PMC5056420; doi:10.1038/ncomms12993)
Supplement: Supplementary Information — Supplementary Figures 1-5 and Supplementary Tables 1-2 [file ncomms12993-s1.pdf]

Supplementary Information

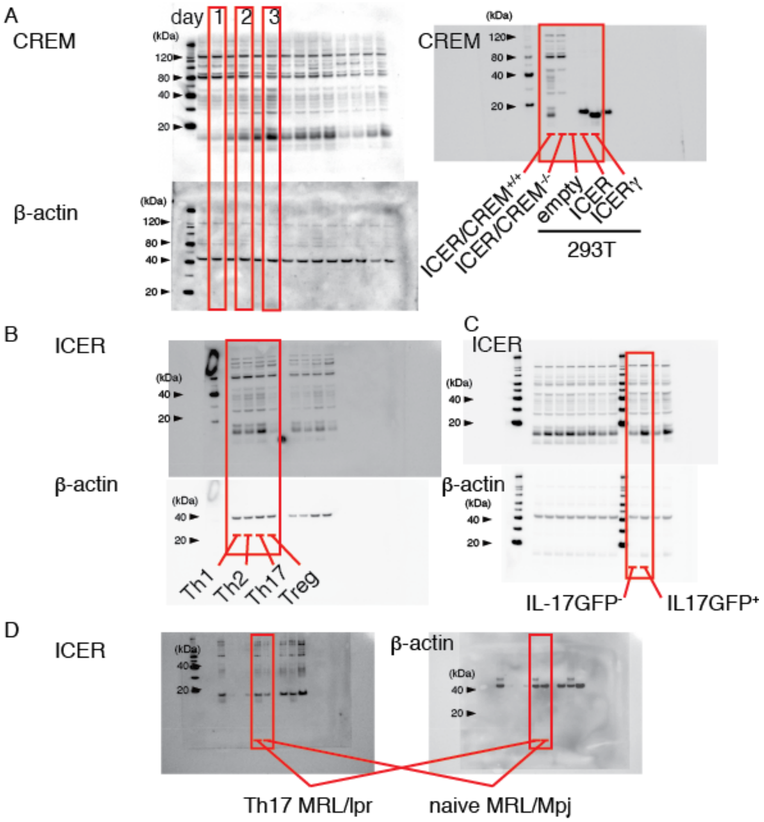

Supplementary Figure 1. Uncropped scans of the western blots shown in Figure 1.

Each figure (A-D) represents the uncropped scan of the western blots including marker positions shown in Figure 1A-D, respectively. Band images shown in Figure 1 are marked in red.

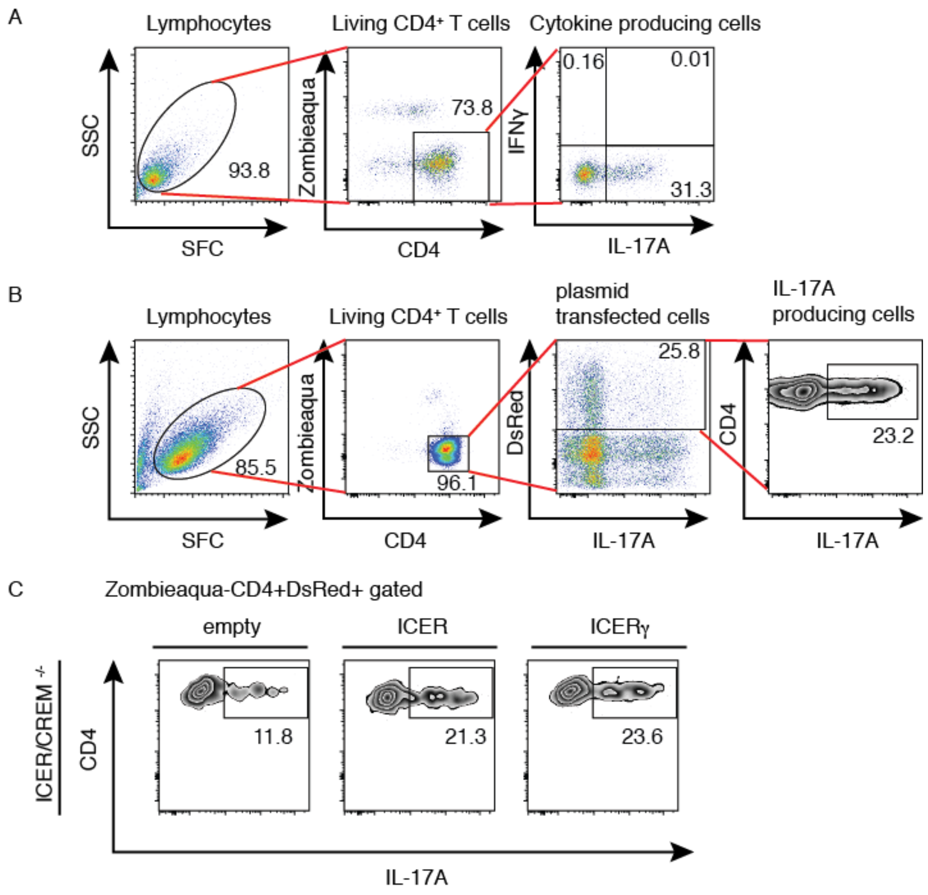

Supplementary Figure 2.

(A) The gating strategy used in Figure 2A to define 7AAD-CD4$^+$ subsets. (B) The gating strategy used in Figure 2G and Supplementary Figure 2C to define Zombieaqua$^-$ CD4$^+$DsRed$^+$ subsets. (C) IL-17A expression after FLAG-ICER plasmid transfection. Empty vector (Empty), FLAG-tagged-ICER expressing (ICER), or FLAG-tagged-ICERγ expressing (ICERγ) plasmids were transfected to Th17 polarized T cells from ICER/CREM$^{-/-}$ IL-17A reporter mice on day1, and the percentage of IL-17 producing cells in was measured by flow cytometry. Data is representative in two experiments.

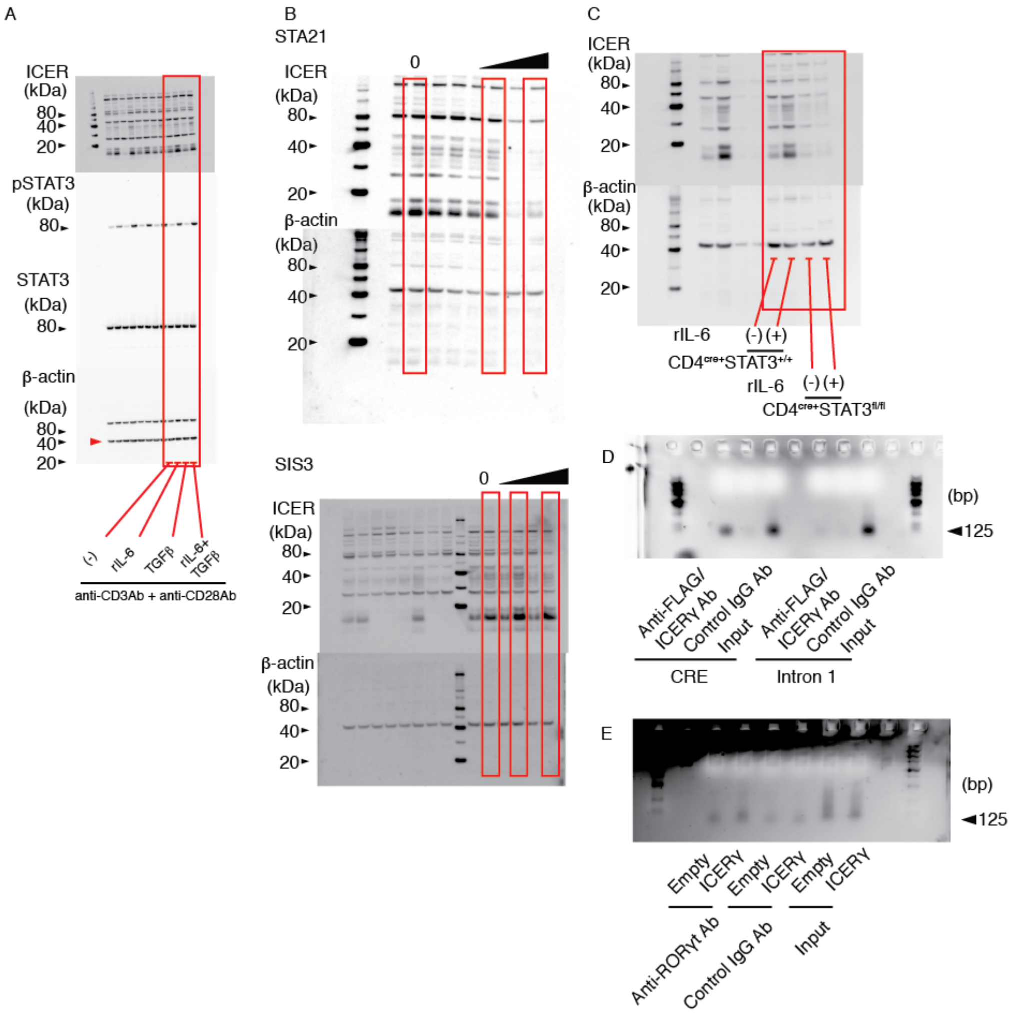

Supplementary Figure 3. Uncropped scans of the western blots shown in Figure 3.

(A-C) Each figure represents the uncropped scan of the western blots including marker positions shown in Figure 3A-C, respectively. (F and I) The uncropped scan of amplicons followed by ChIP assay including marker positions shown in Figure 3F and I, respectively. Band images shown in Figure 3 are marked in red.

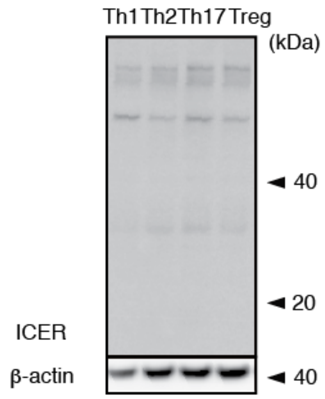

Supplementary Figure 4. ICER expression in unstimulated human memory T cells.

ICER and β-actin expression in FACS sorted Th1, Th2, Th17, and Treg cells were determined by western blot. Data is representative in three experiments. See Supplementary Figure 5 for uncropped scans of the western blot.

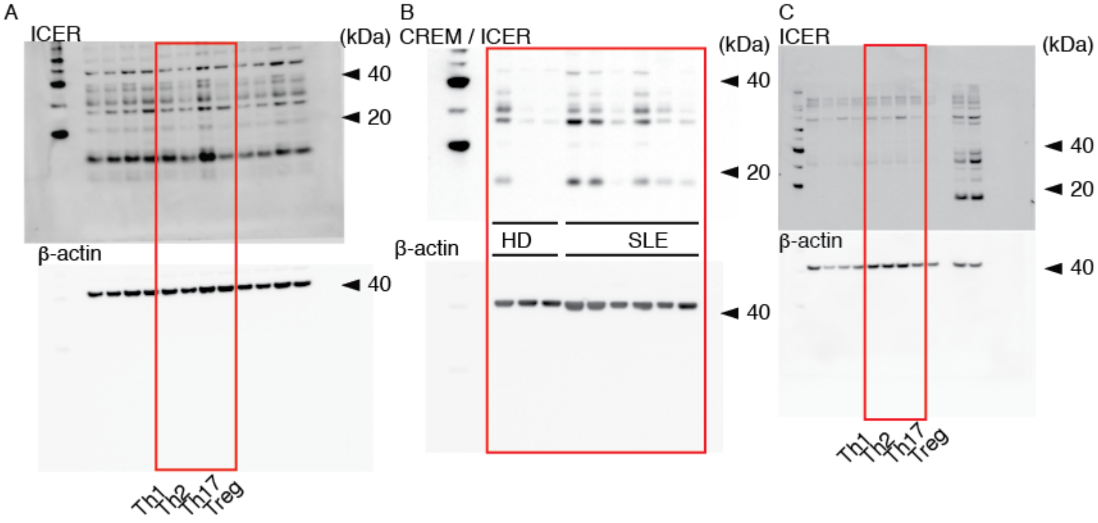

Supplementary Figure 5. Uncropped scans of the western blots shown in Figure 7 and Supplementary Figure 4. Each figure (A-C) represents the uncropped scan of the western blots including marker positions shown in Figure 7B, 7C and Supplemental Figure 4, respectively. Band images shown in Figure 7 and Supplementary Figure 4 are marked in red.

SLE	Age (year)	Sex	Ethnicity	SLEDAI
n=17	mean±median 34.4±2.7			mean±median 4.4±1.6
Lupus 1	46	Female	Black	0
Lupus 2	58	Female	White	13
Lupus 3	50	Female	White	2
Lupus 4	40	Male	Hispanic	3
Lupus 5	27	Female	Asian	0
Lupus 6	22	Female	Black	0
Lupus 7	29	Male	Asian	3
Lupus 8	35	Female	White	2
Lupus 9	33	Male	Asian	16
Lupus 10	27	Female	White	2
Lupus 11	46	Female	Black	4
Lupus 12	24	Female	White	1
Lupus 13	21	Female	Asian	22
Lupus 14	34	Male	Black	0
Lupus 15	20	Female	Asian	6
Lupus 16	43	Female	Black	0
Lupus 17	30	Female	Hispanic	0

Healthy Donors			
n=9	mean±median 42.56±4.1		
HD 1	56	Female	Black
HD 2	44	Female	Black
HD 3	42	Male	White
HD 4	62	Female	White
HD 5	28	Female	Hispanic
HD 6	47	Female	Asian
HD 7	38	Female	Asian
HD 8	22	Female	Asian
HD 9	44	Female	Black

Supplementary Table 1. Demographic information for human subjects.

Demographic information for human subjects included in Figure 7C is shown.

Il17a	5’-TCC AGA AGG CCC TCA GAC TA-3’<br><br>5’-AGC ATC TTC TCG ACC CTG AA-3’
Il17f	5’-CCC CAT GGG ATT ACA ACA TC-3’<br><br>5’-GGA GCA TCT TCT CCA ACC TG-3’
Il23r	5’-GGT CTT CTT GGC CAT CAT GT-3’<br><br>5’-GCC ACT TTG GGA TCA TCA GT-3’
Foxp3	5’-AGG CCC TTC TCC AGG ACA GA-3’<br><br>5’-GCT GAT CAT GGC TGG GTT GT-3’
Tbet	5’-TCA ACC AGC ACC AGA CAG AG-3’<br><br>5’-AAA CAT CCT GTA ATG GCT TGT G-3’
Gata3	5’-TTA TCA AGC CCA AGC GAA G-3’<br><br>5’-TGG TGG TGG TCT GAC AGT TC-3’
Rorγt	5’-ACC TCT TTT CAC GGG AGG A-3’<br><br>5’-TCC CAC ATC TCC CAC ATT G-3’

Supplementary Table 2. Oligonucleotides information for quantitative PCR.

Following primers were used for detecting each gene expression.
